# Persistent postoperative step-off of the posterior malleolus leads to higher incidence of post-traumatic osteoarthritis in trimalleolar fractures

**DOI:** 10.1007/s00402-018-3056-0

**Published:** 2018-11-14

**Authors:** Samuel Marinus Verhage, Pieta Krijnen, Inger Birgitta Schipper, Jochem Maarten Hoogendoorn

**Affiliations:** 1 Department of Surgery, Haaglanden Medical Center, Lijnbaan 32, The Hague, VA 2512 The Netherlands; 2 Department of Traumatology Surgery, Leiden University Medical Center, Leiden, The Netherlands

**Keywords:** Ankle fracture, Posterior malleolar fracture, Trimalleolar fracture, Osteosynthesis, Posterolateral approach

## Abstract

**Background:**

Traditionally, size of the posterior fragment is considered the most important indicator for fixation in trimalleolar fractures. It remains unclear which factors contribute to worse functional and radiological outcome. This study was designed to determine predictors for the development of posttraumatic osteoarthritis and worse functional outcome in trimalleolar fractures.

**Methods:**

This retrospective cohort study evaluated outcomes of 169 patients with a trimalleolar fracture treated between 1996 and 2013 in a level-1 trauma hospital in the Netherlands after a mean follow-up of 6.3 (range 2.4 to 15.9) years. The average fragment size was 17%. Twenty patients had a posterior fragment smaller than 5% of the intra-articular surface, 119 patients a fragment of 5–25% and 30 patients a posterior fragment larger than 25%. In total, 39 patients (23%) underwent fixation of the posterior fragment.

**Results:**

Clinical union was achieved in all 169 patients. The median AOFAS score after follow-up was 93 (interquartile range 76–100) and the median AAOS score was 92 (interquartile range 81–98). A persistent postoperative step-off larger than 1 mm was found in 65 patients (39%) and osteoarthritis was present in 49 patients (30%). Higher age and postoperative step-off > 1 mm were independent, significant risk factors for the development of osteoarthritis. Osteoarthritis and BMI were independent, significant risk factors for worse functional outcome.

**Conclusion:**

It is advisable to correct intra-articular step-off of intraarticular posterior malleolar fragments to reduce the risk of developing osteoarthritis and, consequently, the risk of worse functional outcome after long-term follow-up.

**Level of evidence:**

Level IIB.

## Introduction

The optimal treatment of the posterior malleolus in trimalleolar fractures is a matter of debate amongst orthopaedic trauma surgeons. According to several studies, a fracture of the posterior malleolus has a negative influence on the functional outcome [1–4]. Traditionally, the size of the posterior malleolar fracture fragment is considered the most important indicator for fixation. This is based on the results of biomechanical cadaver studies and several retrospective cohort studies [1, 5–10]. Recent studies showed that both the postoperative position of the posterior malleolar fragment and the postoperative joint congruency are important predictors for functional outcome [11–13]. According to the current guidelines of the Arbeitsgemeinschaft für Osteosynthesefragen (AO) for the treatment of posterior malleolar fragments, fixation of the posterior fragment is not necessary if the fragment is smaller than 25% of the involved intra-articular surface AND if the joint is stable after fixation of the lateral and medial malleolus. Fixation with lag screws is advised if the posterior fragment is larger than 25% of the involved intra-articular surface and long proximal extension is absent. The use of an additional buttress plate is advised in case of a large posterior fragment (> 25%) and long proximal extension. Recently, three CT-based classification systems of posterior malleolar fractures were developed [14–16]. With increasing stage the need for anatomical reduction and fixation increases to restore intra-articular unevenness. Several authors, therefore, advocate a lower threshold for fixating the posterior malleolus [16–18]. The recommendations in these studies however are based on CT-scans only and not on patient-reported functional outcome. Due to changing indications, the fixation of the posterior fragment via a posterolateral approach is gaining popularity [12, 19–21]. Open reduction and internal fixation of the posterior fragment are assumed to reduce intra-articular step-off better than reduction by ligamentotaxis with additional percutaneous anterior-to-posterior screw fixation [12, 17, 18]. In addition, the posterolateral approach enables the removal of intra-articular loose fragments that may interfere with anatomical reduction.

As stated above, no clear guidelines based on large clinical studies about when and how to fixate the posterior fragment in trimalleolar fractures are available. This large retrospective study was designed to determine predictors for the development of posttraumatic osteoarthritis and worse functional outcome in trimalleolar fractures.

## Materials and methods

We conducted a retrospective study of all patients with a posterior malleolar fracture who were operatively treated between 1996 and 2013 in a level 1 trauma hospital (Haaglanden Medical Center, The Hague, The Netherlands) with a follow-up of minimally two years. Since 2011, we gradually changed our operative strategy to an open reduction and internal fixation via the posterolateral approach.

The study was approved by the institutional medical ethics review board (protocol number NL55397.098.15). All patients provided written informed consent before study participation.

Only patients treated with open reduction and internal fixation of an isolated trimalleolar fracture older than 18 years at date of trauma and younger than 75 years at date of follow-up were included. Patients with impaired ankle function prior to injury were excluded. Also patients with an isolated injury of the posterior malleolus were excluded.

All 327 eligible patients in the study period 1996–2013 were invited to participate by letter. Patients who did not respond were contacted by phone. If contact data were missing or outdated, we tried to acquire the recent contact data via the patient’s general practitioner. Patients who agreed to participate in the study were physically examined at the outpatient clinic. Range of Motion, ankle stability and stance of both feet were measured. The difference in dorsiflexion between the two ankles was noted as dorsiflexion restriction. In addition the patients completed a questionnaire and lateral, AP and mortise X-rays were performed.

**Fig. 1:**
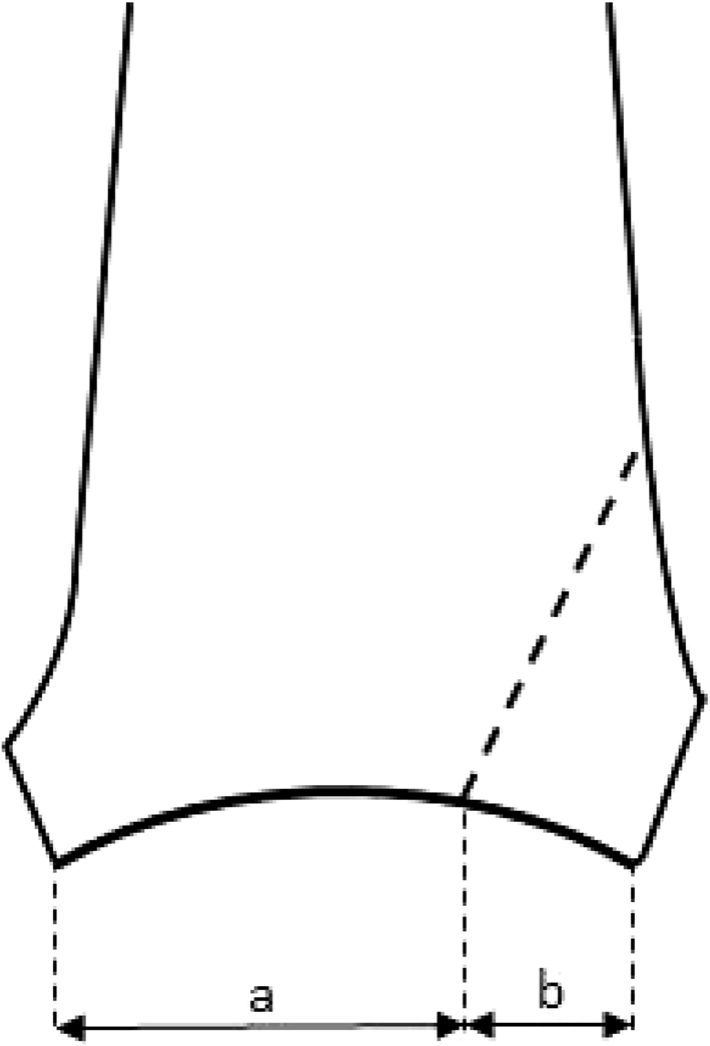
Measurement of posterior malleolus on lateral radiograph, with size defined as: *b*/(*a* + *b*)

All fractures were classified according to the AO- and Lauge-Hansen classification by two independent observers. All fractures had both lateral and posterior malleolar fractures and, in most cases, also a medial malleolar fracture. Posterior fragment size was measured on the lateral preoperative X-ray (Fig. 1). Persistent postoperative articular step-off and gap were measured on postoperative lateral X-rays. Anatomical reduction was defined as a step-off or gap of 1 mm or less. Post-traumatic osteoarthritis was measured on X-ray at time of follow-up and classified according to Domic et al. [19], with 0 indicating a normal joint, 1 indicating osteophytes without joint space narrowing, 2 indicating presence of osteophytes with joint space narrowing and 3 indicating severe joint space narrowing or absence of joint space.

The AAOS (American Academy of Orthopedic Surgeons Foot and Ankle questionnaire) [20] and AOFAS (American Orthopaedic Foot and Ankle Society Ankle-Hindfoot scale) [21] questionnaires were used to assess functional outcome. Both questionnaires are scored on a scale from 0 (worst possible function) to 100 (best possible function). A Visual Analogue Scale (VAS) was used to assess pain at time of follow-up on a scale from 0 (no pain) to 10 (worst imaginable pain).

IBM SPSS Statistics for Windows version 22 (IBM Corp., Armonk, NY, USA) was used for data entry and statistical analysis. Baseline and outcome data of the study group were described using summary statistics. To identify risk factors for the development of osteoarthritis, logistic regression analysis was performed with age, BMI, time to follow-up, fragment size, fragment fixation, postoperative step-off > 1 mm and postoperative gap > 1 mm as independent variables. To identify risk factors for a poor functional outcome, linear regression analyses were performed with the same patient-related factors and presence of osteoarthritis as independent variables and functional outcome (AAOS and AOFAS) as dependent variables. First, univariable regression analyses were performed. Then, the factors with a univariable association of *p* < 0.20 with the outcome were combined in the multivariable analysis. *p*-values < 0.05 were considered statistically significant.

## Results

### Patient characteristics

In this study, 169 patients (52% of the 327 eligible patients) were evaluated at the outpatient clinic with a mean follow-up of 6.3 years (range 2.4–15.9 years). The characteristics at baseline are presented in Table 1. The average fragment size was 17% with range from 3 to 44%. Twenty patients (12%) had a posterior fragment smaller than 5% of the intra-articular surface, 119 patients (70%) a fragment between 5 and 25% and 30 patients (18%) a posterior fragment larger than 25%. In total, 39 patients (23%) underwent fixation of the posterior fragment, 23 patients of whom underwent open reduction and internal fixation via a posterolateral approach. None of the posterior fragments with a size < 5% were fixated, whereas this was done in 15 (13%) of the 5–25% sized fragments and in 24 (80%) of the fragments with a size > 25% (*p* < 0.0001). Fixation took place in 39 cases (23%). Anatomical reduction of the posterior fragment was only achieved in 23 out of 39 patients (69%; ORIF 7/23; AP 9/16). If persistent syndesmotic instability was present after fixation, a syndesmotic positioning screw was placed to stabilize the distal tibiofibular syndesmosis. All fractures were clinically stable after fixation.

**Table 1:** Patient characteristics

Number of patients	169
Follow-up in years, mean (range)	6.3 (2.4–15.9)
Age in years, mean (SD)	52.3 (13.0)
Male, n (%)	67 (39.6)
Smoking, *n* (%)	41 (24.3)
Diabetes (*n*, %)	12 (7.1)
BMI, mean (SD)	27.6 (5.2)
Lauge-Hansen classification, *n* (%)	
PA3	9 (5.3)
SE3	6 (3.6)
SE4	106 (62.7)
PE4	48 (28.4)
AO classification, *n* (%)	
AO-44B	122 (72.2)
AO-44C	47 (27.8)
PMFF size in %, mean (SD)	16.9 (10.1)
PMFF size, *n* (%)	
< 5%	20 (11.8)
5–25%	119 (70.4)
> 25%	30 (17.8)
Medial malleolus fracture, *n* (%)	121 (71.6)
PMFF fixation, *n* (%)	
No fixation	130 (76.9)
Fixation ‘A to P’	16 (9.5)
ORIF	23 (13.6)

*SD* standard deviation, *BMI* body mass index, *PMFF* posterior malleolar fracture fragment, *ORIF* open reduction and internal fixation

### Clinical outcome

Clinical union was achieved in all 169 patients. A persistent postoperative step-off larger than 1 mm was found in 65 patients (39%) of the cases and a postoperative gap larger than 1 mm was found in 58 (35%) of the cases. Osteoarthritis was present in 49 patients (30%), no re-operations due to osteoarthritis were performed during the follow-up period. The median AOFAS score after follow-up was 93 (interquartile range [IQR] 76–100) and the median AAOS score was 92 (IQR 81–98) (Table 2). The median pain score after follow-up was 9 (IQR 0–25).

**Table 2:** Functional and radiological outcome

AOFAS, median (IQR)	93 (76–100)
AAOS, median (IQR)	92 (81–98)
VAS pain, median (IQR)	9 (0–25)
Dorsiflexion restriction in, median (IQR)	5 (0–10)
Step-off > 1 mm, *n* (%)	65 (39)
Gap > 1 mm, *n* (%)	58 (35)
Osteoarthritis grade, *n* (%)	
0	113 (70)
1	23 (14)
2	18 (11)
3	8 (5)

*IQR* interquartile range

### Risk factors for osteoarthritis

In the univariable analyses, age, fragment size, fragment fixation and postoperative step-off > 1 mm and gap > 1 mm were potential risk factors for osteoarthritis (*p* < 0.20; Table 3), and were entered in the multivariable logistic regression model. Two independent and statistically significant risk factors for development of osteoarthritis were identified: higher age (odds ratio [OR] per one-year increase 1.03, 95% confidence interval [CI] 1.001 to 1.06, *p* = 0.04) and postoperative step-off > 1 mm (OR 4.16, 95% CI 1.50–11.57, *p* = 0.006). Fragment size, fragment fixation and postoperative gap > 1 mm were not associated with osteoarthritis after follow-up in the multivariable model (*p* > 0.05; Table 3).

**Table 3:** Risk factors for development of osteoarthritis

Factor	Univariable analysis	Multivariable analysis
Age (years)	1.04 (1.01 to 1.07; *p* = 0.01)	1.03 (1.00 to 1.06, *p* = 0.04)
BMI	1.02 (0.95 to 1.10, *p* = 62)	(Not entered)
Time to follow-up (years)	1.03 (0.94 to 1.14, *p* = 0.50)	(Not entered)
PMFF size (%)	1.03 (1.00 to 1.07, *p* = 0.05)	1.00 (0.96 to 1.05, *p* = 0.95)
PMFF fixation	2.03 (0.95 to 4.32, *p* = 0.07)	2.09 (0.70 to 6.29, *p* = 0.19)
Postoperative step-off > 1 mm	3.27 (1.63 to 6.56, *p* = 0.001)	4.16 (1.50 to 11.57, *p* = 0.006)
Postoperative gap > 1 mm	1.82 (0.91 to 3.62, *p* = 0.09)	0.80 (0.28 to 2.26, *p* = 0.67)

Results are presented as odds ratio with 95% confidence interval and p-value

### Risk factors for worse functional outcome

In the univariable analyses, BMI, fragment size and presence of osteoarthritis were potential risk factors (*p* < 0.20) for lower scores for both the AAOS and AOFAS. Fragment fixation was a potential risk factor for lower AOFAS scores, but not for lower AAOS scores (Table 4). In the multivariable analyses, the presence of osteoarthritis was a statistically significant risk factor for a worse functional outcome: osteoarthritis was associated with a 12.55 points lower AAOS score (95% CI 5.99–19.11, *p* < 0.0001) and with a 15.43 points lower AOFAS score (95% CI 8.06–22.80, *p* < 0.0001). BMI was a statistically significant risk factor for worse AAOS score: a 1-point increase in BMI was associated with a decrease of 0.77 points on the AAOS scale (95% CI 0.21–1.33, *p* = 0.008). In the multivariable model for AOFAS, BMI was borderline significant (*p* = 0.06, Table 4).

**Table 4:** Risk factors for worse functional outcome measured with (A) AAOS and (B) AOFAS

Factor	Univariable analysis	Multivariable analysis
(A) AAOS		
Age (years)	− 0.06 (− 0.26 to 0.13; *p* = 0.53)	(Not entered)
BMI	− 0.80 (− 1.39 to − 0.20, *p* = 0.009)	− 0.77 (− 1.33 to − 0.21, *p* = 0.008)
Time to follow-up (years)	− 0.05 (− 0.42 to 0.09, *p* = 0.89)	(Not entered)
PMFF size (%)	− 0.17 (− 0.51 to 0.08, *p* = 0.19)	− 0.16 (− 0.46 to 0.14, *p* = 0.28)
PMFF fixation	− 3.78 (− 9.96 to 2.40, *p* = 0.23)	(Not entered)
Postoperative step-off > 1 mm	− 2.68 (− 7.99 to 2.63, *p* = 0.32)	(Not entered)
Postoperative gap > 1 mm	− 0.37 (− 5.84 to 5.11, *p* = 0.90)	(Not entered)
Presence of osteoarthritis	− 12.55 (− 17.85 to − 7.25, *p* < 0.0001)	− 12.55 (− 19.11 to − 5.99, *p* < 0.0001)
(B) AOFAS		
Age (years)	− 0.08 (− 0.31 to 0.15; *p* = 0.49)	(Not entered)
BMI	− 0.66 (− 1.35 to 0.03, *p* = 0.06)	− 0.62 (− 1.27 to 0.02, *p* = 0.06)
Time to follow-up (years)	− 0.36 (− 1.24 to 0.52, *p* = 0.43)	(Not entered)
PMFF size (%)	− 0.22 (− 0.51 to 0.08, *p* = 0.15)	− 0.17 (− 0.60 to 0.26, *p* = 0.43)
PMFF fixation	− 6.54 (− 13.58 to 0.50, *p* = 0.07)	− 1.52 (− 12.34 to 9.30, *p* = 0.78)
Postoperative step-off > 1 mm	− 3.18 (− 9.22 to 2.87, *p* = 0.30)	(Not entered)
Postoperative gap > 1 mm	− 1.87 (− 8.05 to 4.31, *p* = 0.55)	(Not entered)
Presence of osteoarthritis	− 15.89 (− 21.94 to − 9.85, *p* < 0.0001)	− 15.43 (− 22.80 to − 8.06, *p* < 0.0001)

Results are presented as difference in functional outcome score with 95% confidence interval and *p* value

## Discussion

Optimal treatment of trimalleolar fractures, especially treatment of the posterior malleolus in trimalleolar fractures remains a matter of debate amongst orthopaedic trauma surgeons. For a long period, the size of the posterior fragment was the decisive factor for reduction and fixation of the fragment. Traditionally, this is done for fragments larger than one quarter or one-third of the articular surface via ligamentotaxis followed by percutaneous anterior-to-posterior screw placement. In clinical practice, however, it is difficult to estimate the size of the posterior fragment on lateral photographs and even on 2D CT-scans [14, 16, 22, 23].

This retrospective study showed that postoperative step-off (> 1 mm) and age were independent risk factors for the development of osteoarthritis. The presence of osteoarthritis and a high BMI were identified as independent and significant risk factors for worse functional outcome. Notably, both the size and the fixation of the posterior fragment were associated neither with the development of osteoarthritis nor with functional outcome. Since postoperative intra-articular step-off is an independent risk factor for osteoarthritis irrespective of fragment size on lateral X-rays, it seems advisable to anatomically reduce and fixate all intra-articular posterior fragments.

We already know from previous studies that fractures without articular involvement do not need anatomical reduction and fixation. Anatomical reduction and fixation of these fragments do not improve posterior stability and the risk of osteoarthritis is comparable with uni- or bimalleolar fractures without involvement of the posterior malleolus [5, 9]. Fixation of intra-articular posterior malleolar fractures was not identified as an independent protective factor for an adverse outcome in this study. Probably because of the 16 anterior to posterior fixated fragments, which leads to a higher risk of non-anatomical reduction. Since a few years, the posterolateral approach is gaining more and more popularity amongst orthopaedic surgeons. An anatomical reduction and stable fixation via this approach is more easily to achieve and leads to a reduction of persistent postoperative step-off [24, 25]. A postoperative gap (> 1 mm) was not identified as independent risk factor for an adverse outcome in our analysis. Closing the gap seems, therefore, less important than correction of the postoperative step-off.

The strongest point of this study is the large cohort with a mean follow-up time of more than 6 years. Up to now, this is the largest cohort of trimalleolar fractures describing functional outcome and development of osteoarthritis as outcome parameters. Previously published studies on this topic included smaller cohorts [1, 3, 7, 8, 11, 13] or described a shorter follow-up period [26–28] which makes it more difficult to draw strong conclusions.

A limitation of this study is the relatively large part of the eligible patients were lost to follow-up or did not want to participate in the study. In addition, the retrospective design of this study may have led to an observer bias. Another important limitation concerns the measurement of posterior malleolar fragment size on lateral preoperative X-rays and the measurement of postoperative step-off on lateral X-rays. Some articles that were published in the last few years have reported that the interobserver agreement of fragment size on lateral X-rays is poor [29, 30] and even CT-scanning is not reliable to compare different fragment sizes [22, 23]. Therefore, the possible relation between fragment size and functional outcome may have been missed in our study because of inaccurate measurements. In our opinion, however, the measurement on lateral X-rays is good enough to distinguish a truly intra-articular fracture from a small extra-articular fracture. Other studies suggest an important role of fracture localization and geometry instead of fracture size as diagnosed on CT-scan [14–16, 31]. Anatomical reduction and posterior fragment fixation is advocated for nearly all posterior fractures except the small extra-incisural fractures [31, 32]. These recommendations however are only derived from CT-based studies without any functional outcome. Unfortunately, we could not relate these different posterior fracture characteristics due to lack of a large part of pre-operative CT-scans.

Clear indications for fixation of the posterior malleolus based on clinical studies are lacking in the current literature. With respect to the size of the posterior malleolus, the cut-off value above which the posterior fragment should be fixated is at least inconclusive. A number of biomechanical cadaveric studies have been performed without providing one clear conclusion [5, 6, 9, 10, 33]. Comparative clinical studies are also inconsistent in their results. Most authors of clinical studies agree that operative treatment is indicated for large posterior fragments and prefer conservative treatment in small avulsion fractures [1, 7, 11, 13, 14, 24]. Although there is some evidence that open reduction and internal fixation of posterior fragments up to 25% lead to better results than conservative treatment, this statement is at least controversial [1, 7, 8, 11, 13, 27]. All of these studies have limitations due to their retrospective design. On the basis of these publications the AO advises fixation of the posterior fragment only in case of a large fragment (> 25%) or for smaller fragments with persistent instability after fixation of both the lateral and medial malleolus.

This largest retrospective study involving trimalleolar fractures up to now suggests that size of the posterior fragment is not an independent risk factor for both development of osteoarthritis and functional outcome. Postoperative step-off is an important risk factor that can be influenced to reduce the risk of post-traumatic osteoarthritis and consequently increase the probability of a good functional outcome on the long term. In addition, reducing obesity may help to improve functional outcome. Open reduction and internal fixation via the posterolateral approach leads to a decrease in postoperative step-off compared to closed reduction through ligamentotaxis followed by anterior to posterior screw fixation [12, 17, 18]. It is, therefore, advisable to treat posterior fragments via a posterolateral approach to correct intra-articular step-off. At this moment the POSTFIX study (RCT) is enrolled in our hospitals to prove this statement [34].

## Conclusion

Increasing age and persistent postoperative step-off are independent risk factors in the development of post-traumatic osteoarthritis in patients with trimalleolar fractures. In turn, development of osteoarthritis as well as high BMI are associated with worse functional outcome. Of these, persistent postoperative step-off is the only risk factor that can be influenced by surgeons. Therefore, it is advisable to correct intra-articular step-off of intra-articular posterior malleolar fragments.
